# m5C-Related Signatures for Predicting Prognosis in Cutaneous Melanoma with Machine Learning

**DOI:** 10.1155/2021/6173206

**Published:** 2021-08-04

**Authors:** Maoxin Huang, Yi Zhang, Xiaohong Ou, Caiyun Wang, Xueqing Wang, Bibo Qin, Qiong Zhang, Jie Yu, Jianxiang Zhang, Jianbin Yu

**Affiliations:** ^1^Department of Dermatology, The first Affiliated Hospital of Zhengzhou University, Zhengzhou, Henan 450052, China; ^2^Department of Gastroenterology, The first Affiliated Hospital of Zhengzhou University, Zhengzhou, Henan 450052, China; ^3^Department of Oncology, The first Affiliated Hospital of Zhengzhou University, Zhengzhou, Henan 450052, China

## Abstract

**Background:**

Cutaneous melanoma (CM) is one of the most life-threatening primary skin cancers and is prone to distant metastases. A widespread presence of posttranscriptional modification of RNA, 5-methylcytosine (m5C), has been observed in human cancers. However, the potential mechanism of the tumorigenesis and prognosis in CM by dysregulated m5C-related regulators is obscure.

**Methods:**

We use comprehensive bioinformatics analyses to explore the expression of m5C regulators in CM, the prognostic implications of the m5C regulators, the frequency of the copy number variant (CNV), and somatic mutations in m5C regulators. Additionally, the CM patients were divided into three clusters for better predicting clinical features and outcomes via consensus clustering of m5C regulators. Then, the risk score was established via Lasso Cox regression analysis. Next, the prognosis value and clinical characteristics of m5C-related signatures were further explored. Then, machine learning was used to recognize the outstanding m5C regulators to risk score. Finally, the expression level and clinical value of USUN6 were analyzed via the tissue microarray (TMA) cohort.

**Results:**

We found that m5C regulators were dysregulated in CM, with a high frequency of somatic mutations and CNV alterations of the m5C regulatory gene in CM. Furthermore, 16 m5C-related proteins interacted with each other frequently, and we divided CM patients into three clusters to better predicting clinical features and outcomes. Then, five m5C regulators were selected as a risk score based on the LASSO model. The XGBoost algorithm recognized that NOP2 and NSUN6 were the most significant risk score contributors. Immunohistochemistry has verified that low expression of USUN6 was closely correlated with CM progression.

**Conclusion:**

The m5C-related signatures can be used as new prognostic biomarkers and therapeutic targets for CM, and NSUN6 might play a vital role in tumorigenesis and malignant progression.

## 1. Introduction

Cutaneous melanoma (CM) is one of the most common, aggressive, and life-threatening types of malignant primary skin cancers and is prone to distant metastases associated with a high mortality rate [1, 2]. Surgical management, chemotherapy agents, and immunotherapy have been considered the fundamental treatment of CM in recent years [3, 4]. Although great achievements have been made in CM treatment, the 5-year overall survival with metastatic melanoma patients remains poor, attributed to late diagnosis [5, 6], rapid metastasis, and poor response to treatment [7, 8]. Therefore, exploring powerful prognostic predictors and a novel therapeutic target is urgent and crucial to improving CM diagnosis and treatment.

Additionally, 5-methylcytosine (m5C) is a widespread in posttranscriptional modification of RNA which has been observed in substantial RNA species, including rRNAs and tRNAs, mRNAs, eRNAs, and noncoding RNAs in the cytoplasm and mitochondria [9–18]. The methylation of m5C involves a series of regulators, including m5C methyltransferases, demethylases, and “readers.” The methyltransferase “writer,” including NSUN1-7, DNMT1, DNMT2, DNMT3A, and DNMT3B, increases methylation at the C5 position of RNAs. Then, different “reader” proteins, such as TET1-3, recognize and bind the methylated mRNAs, while the “eraser” protein, such as ALYREF and YBX1, reverses m5C modification by degrading the written methylation [19, 20]. Some studies have indicated that m5C modification plays an important role in many biological functions, including ribosome assembly, tRNA stability, mRNA export, protein transcription, and stem cell regulation [21, 22]. It has been reported that the m5C gene mutations are closely associated with various human diseases such as nervous system disorders, metabolic disease, and virus infections [23, 24]. In addition, dysregulated m5C regulators have been observed in human cancers such as breast, gallbladder, and bladder cancer [25–28]. However, the potential tumorigenesis mechanism and prognosis in CM by dysregulated m5C-related regulators is obscure.

In this study, we explored the mRNA level of expression of m5C regulators by using The Cancer Genome Atlas (TCGA) database and Gene Expression Omnibus (GEO) database. Then, we analyzed the frequency of m5C regulators' copy number variant (CNV) and somatic mutations. The CM patients were divided into three clusters to better predict clinical features and outcomes via consensus clustering of m5C regulators. The established prognostic gene signature showed a five-gene prognostic signature, which had an effective prediction ability of the progression and prognosis of CM patients, comprising of NOP2, NSUN3, NSUN6, DNMT2, and YBX1 via Lasso Cox regression analysis. Moreover, the protein expression levels of NSUN6 were significantly upregulated in the CM, associated with the advanced TNM stage. Further survival analysis indicated that NSUN6 could be an independent risk factor for the prognosis of CM patients. NSUN6 expression was significantly related to the pathologic state and TNM staging of CM. These results showed that m5C-regulator-based prognostic signature can be used as new prognostic biomarkers and therapeutic targets for CM. Additionally, NSUN6 might play vital roles in tumorigenesis and malignant progression.

## 2. Materials and Methods

### 2.1. Datasets

The RNA-seq transcriptome data of CM were downloaded from The Cancer Genome Atlas (TCGA, https://tcga-data.nci.nih.gov/tcga) and the Gene Expression Omnibus (GEO, http://www.ncbi.nlm.nih.gov/geo/GSE3189, GSE7553, GSE31909, GSE46517, GSE98394, and GSE114445). The characteristics of these CM patients and normal people are presented in Tables S1 and S2.

### 2.2. Tissue Samples

The 40 tumors and 8 normal tissue microarrays were purchased from Shaanxi Avila Biotechnology Co., Ltd. (Xi'an, China). Also, our studies were approved by the Ethics Committee of the First Affiliated Hospital of Zhengzhou University.

### 2.3. Immunohistochemistry (IHC)

Immunohistochemistry was performed as previously reported [29, 30]. Semiquantification analysis of immunohistochemical staining was performed using ImageJ (NIH, Bethesda, MD). Two experienced pathologists who were blinded to the clinicopathological data evaluated the immunostaining samples separately, and they scored the samples according to the proportion of positive cells as follows: no staining, 1+; weak staining, 2+; moderate staining, 3+; and strong staining, 4+.  Table S3 lists the information of antibodies used in this study.

### 2.4. Consensus Clustering

Consensus clustering was based on the m5C regulators' profiles in CM using the *R* package “Consensus Cluster Plus,” and to guarantee the stability of clustering, 1000 times repetitions were performed. The appropriate number of clusters was calculated via the cumulative distribution function (CDF) and consensus matrices.

### 2.5. Pathway Enrichment Analysis

Spearman's analysis was conducted to evaluate the correlation between 16 m5C regulators and their function, and interaction analysis was predicted by the String website (https://string-db.org/). A comprehensive gene function analysis was performed with Metascape (http://metascape.org/). To further explore the potential mechanism of the m5C regulators in CM, we performed the gene set variation analysis (GSVA), the Kyoto Encyclopedia of Genes and Genomes (KEGG), and Gene Ontology (GO) analysis [31].

### 2.6. Construction and Validation of Risk Score

To construct the powerful prognostic factors, the five m5C regulators were selected by the Least Absolute Shrinkage and Selection Operator (LASSO) Cox regression model [32]. The Cox regression analysis was conducted for 16 genes, and 0.2 was set as the cutoff *P* value to prevent omissions. The risk score for each patient in both the training (TCGA) and validation (GSE65904) datasets was calculated using the following formula:

(1) $$\begin{array}{c} \text{risk score}=\sum_{i=1}^{n}\text{Coef}*xi. \end{array}$$

Coefi means the coefficient, and xi is the expression value of the z-score transformation of each selected m5C regulator.

### 2.7. Machine Learning Algorithm

We used the machine learning model, extreme gradient, (XGBoost) with the Shapley additive explanation method to explore the importance of m5C regulators to the risk score.

### 2.8. Statistical Analysis

All statistical analyses were performed in the *R* statistical computing language (*R* version 4.0.4) unless otherwise indicated. The differences between the two independent groups were determined using Student's *t*-test (unpaired, two tailed). The chi-square test and rank-sum test were used for qualitative variables. The Kaplan–Meier overall survival (OS) and progression-free survival (PFS) analyses were performed using the log-rank test based on the median. Univariate and multivariate Cox regression analyses were performed to assess independent prognostic factors. *P* < 0.05 was considered statistically significant (^*∗*^*P* < 0.05, ^*∗∗*^*P* < 0.01, and ^*∗∗∗*^*P* < 0.001).

## 3. Results

### 3.1. Variation and Prognosis Value of m5C Regulators in CM

**Figure 1:**
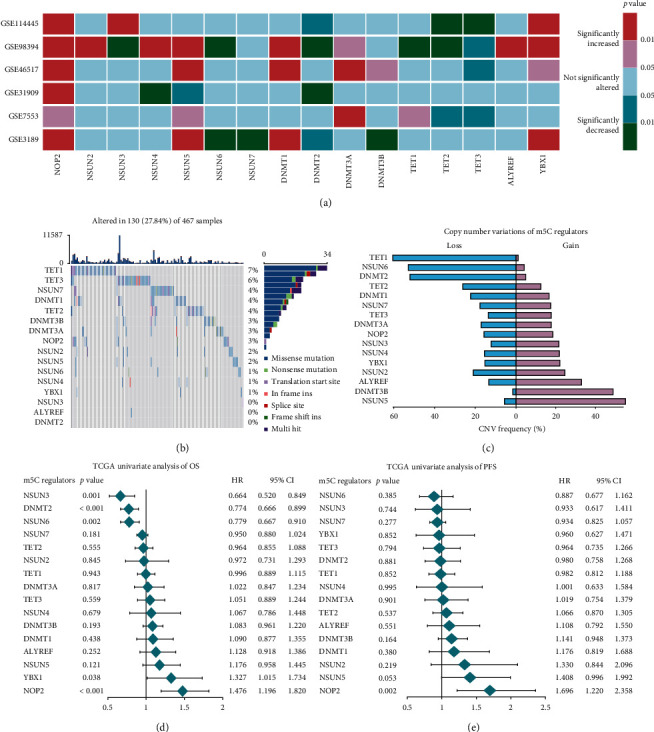
The variation and prognosis value of m5C regulators in CM. (a) The mRNA expression status of m5C regulators in CM. (b) The CNV frequency of m5C regulators in the TCGA-CM cohort. (c) The copy number of 16 m5C-related genes in CM. (d) Univariate Cox analysis of OS in CM. (e) Univariate Cox analysis of PFS in CM.

To probe the significant biological function of m5C regulators in CM, we explore the mRNA expression levels of m5C regulators in CM and normal tissues using public databases. We found that the mRNA expression levels of NOP2, YBX1, DNMT3A, NSUN5, ALYREF, and DNMT1 were significantly upregulated and NSUN6, NSUN7, TET2, and TET3 were significantly decreased in CM tissues (Figure 1(a)). Furthermore, to obtain insight into the causes of dysregulation of m5C regulators, we investigated alterations to the somatic mutations and the CNV alteration frequency of m5C regulators. Among all the 467 patients, 130 experienced m5C regulators mutations with a frequency of 27.84%, indicating a high frequency of somatic mutations of the m5C regulatory gene in CM (Figure 1(b)). We found that the copy number of CNV in NSUN5, DNMT3B, and ALYREF was amplificated while NSUN6, TET1, TET2, and DNMT2 were deleted. NSUN5 and ALYREF consistently represented high mRNA expression levels, while NSUN6 and TET2 indicated low mRNA expression levels (Figure 1(c)). Survival analysis showed that NSUN3, DNMT2, and NSUN6 were potential protective factors and that YBX1 and NOP2 were potential risk factors for OS (Figures 1(d) and S1). Moreover, NOP2 was a potential risk factor for PFS in CM (Figures 1(e) and S2). Overall, as these results presented significant genomic variations of CM m5C regulators, m5C regulators had a potential prognosis value in CM.

### 3.2. Unsupervised Consensus Clustering Analysis of m5C RNA Methylation Modulators

**Figure 2:**
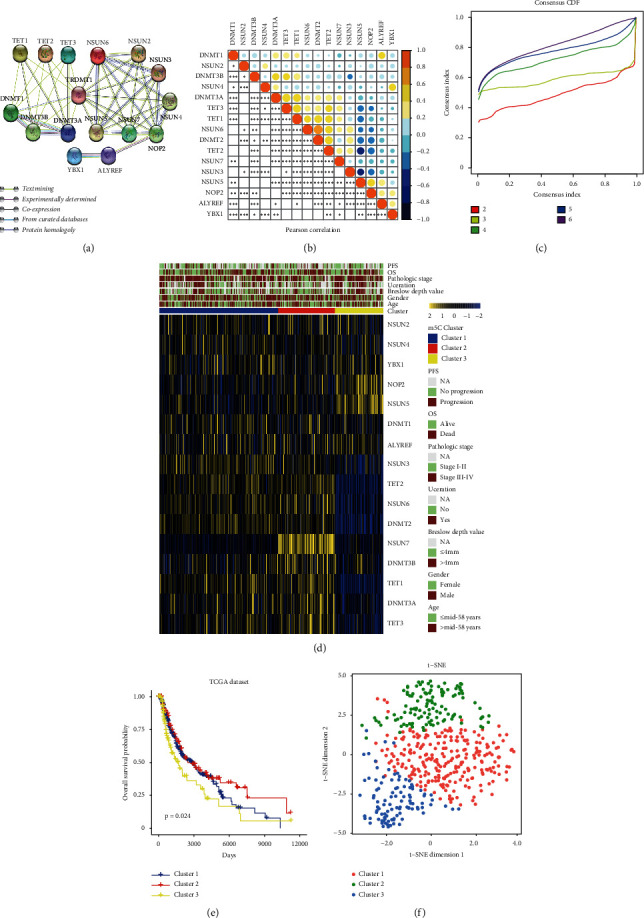
Unsupervised consensus clustering analysis of m5C RNA methylation modulators. (a) The protein-protein interactions of sixteen m5C regulators. (b) Pearson's correlation analysis of the expression of 16 m5C regulators in CM. (c) Consensus clustering matrix for the most suitable groups, *k* = 3. (d) The relationship between the expression levels of m5C regulators and the clinical features in the three clusters of CM. (e) Overall survival analysis for CM patients stratified by the three clusters. (f) t-SNE plots of TCGA-CM RNA-sequence profiles for the three m5C clusters.

To integrally investigate the role of m5C modification in CM, we analyzed the interaction among the 16 m5C-related regulators. Results indicated that the protein-protein interaction networks and m5C-related proteins interacted with each other frequently (Figure 2(a)). Additionally, we explore the correlation among the expression profile of 16 m5C regulators in a TCGA dataset by conducting a Pearson correlation analysis. These results indicated that most m5C regulators were positive correlations, among which the expression of ALYREF, DNMT2, DNMT3B, NSUN5, NSUN6, and TET1 showed a stronger correlation in CM (Figure 2(b)). Thus, there is a close relationship between the biological functions of 16 m5C regulators and CM. Based on the expression levels of the m5C modulators of CM patients, we observed the clustering stability of the TCGA dataset from *k* = 2 to 6 (Figure S3) via unsupervised consensus.

### 3.3. Clustering analysis

Using *k* = 3, we identify these three clusters, designated as m5C cluster 1, m5C cluster 2, and m5C cluster 3 (Figure 2(c) and Table S4). Subsequently, we found that cluster 1 presented moderate expression in most of the m5C regulators and cluster 2 showed the highest expression of USUN6, USUN7, TET1, TET3, DNMT3A, and DNMT3B; cluster 3 exhibited the lowest expression of NSUN3, USUN6, TET1, TET2, TET3, DNMT2, DNMT3A, and DNMT3B (Figure 2(d)). Then, we compared the prognosis between cluster 2 and cluster 3, finding a significant distinction between m5C cluster 2 and cluster 3. According to the prognostic analysis, cluster 2 had a more significant potential survival advantage than cluster 3 (Figure 2(e)). Moreover, a t-SNE dimensional reduction showed that it was feasible to segregate the m5C clusters into three discrete clusters (Figure 2(f)). These results indicate that m5C clusters might be more related to CM prognosis. Next, we compared the clinical features and clinicopathological factors between these three clusters. Chi-square analysis indicated that cluster 3 was significantly associated with higher pathological grade (*P* < 0.05) and deeper Breslow depth value (*P* < 0.001) and cluster 2 was significantly associated with orthotopic tumors, a lower pathological grade, and shallower Breslow depth value (Table S4).

### 3.4. Biological Functional Annotation of m5C Clusters

**Figure 3:**
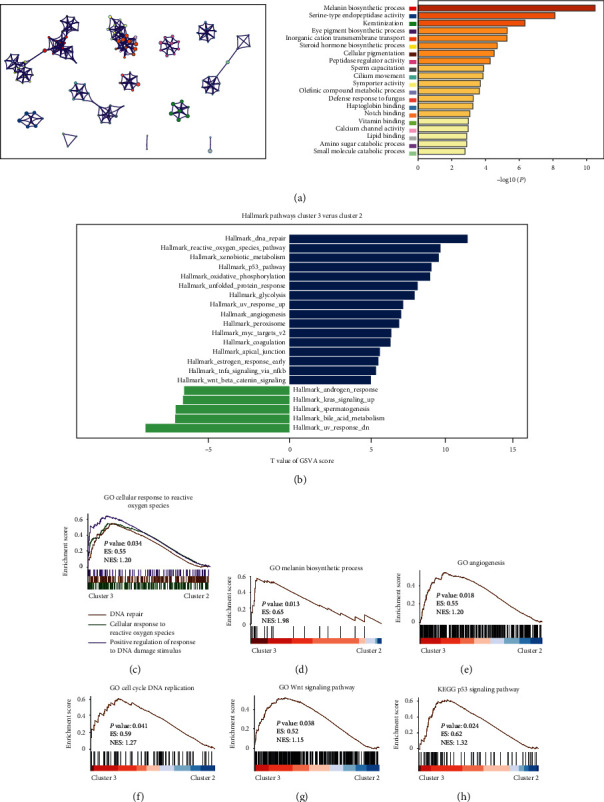
Functional annotation of m5C clusters. (a) The network and the top 20 significantly enriched biological pathways in the m5C clusters. (b) The GSVA between m5C clusters 2 and m5C clusters 3 using hallmark gene sets. (c–h) GSEA showed that the “cellular response to reactive oxygen species,” “melanin biosynthetic process,” “cell cycle DNA replication,” “angiogenesis,” “Wnt signaling pathway,” and “p53 signaling pathway” were significantly enriched in m5C cluster 3.

To further explore the biological behaviors of m5C clusters, we selected the top 500 upregulated expressed genes in cluster 3 based on the *P* value to annotate the biological function. Results indicated that the “Melanin biosynthetic process” and “Keratinization” were significantly enriched in m5C cluster 3. We displayed the top 20 significantly enriched biological processes in Figure 3(a). Simultaneously, the GSVA analysis indicated that certain tumor progression pathways (“DNA repair,” “the reactive oxygen species pathway,” “the p53 signaling pathway,” and “angiogenesis”) were significantly activated in m5C cluster 3 patients (Figure 3(b)). Moreover, GSEA analysis showed that “cellular response to reactive oxygen species,” “the melanin biosynthetic process,” “cell cycle DNA replication,” “angiogenesis,” “the Wnt signaling pathway,” and “the p53 signaling pathway” were significantly enriched in m5C cluster 3, exhibiting worse clinical features and prognosis (Figures 3(c)–3(h)). In summary, there was a significant association between clusters of m5C RNA methylation modulators and the progression of malignant CM.

### 3.5. Prognostic Value and Construction of the Risk Score Signature of m5C Regulators

**Figure 4:**
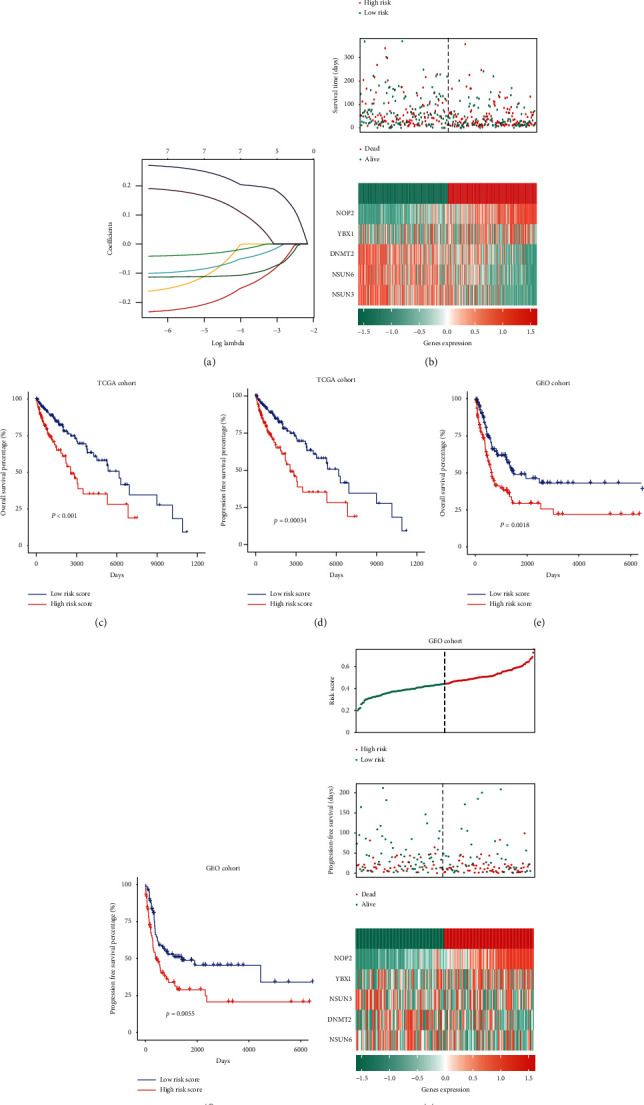
Prognostic value and construction of the risk score signature of m5C regulators. (a) LASSO Cox regression was used to calculate the coefficients for five m5C-related genes. (b) Patients with high-risk scores were correlated with higher death rates among CM patients. (c, d) Kaplan–Meier analysis of OS and PFS in patients with high- and low-risk score groups in the TCGA dataset. (e, f, g) Validation of the risk score signature in the GSE65904 dataset using the same analysis.

We performed a prognostic risk score signature containing DNMT2, NSUN3, NSUN6, YBX1, and NOP2 using a LASSO Cox regression model according to the minimum criterion to evaluate the prognosis value of m5C methylation modification on individual CM patients accurately (Figure 4(a)). The coefficients of DNMT2, NSUN3, NSUN6, YBX1, and NOP2 were −0.01842, −0.08767, −0.07701, 0.00057, and 0.18925, respectively. Thus, we calculated each for CM patients with the formula risk score = (−0.01842 × expression of DNMT2) + (−0.08767 × expression of NSUN3) + (−0.07701 × expression of NSUN6) + (0.00057 × expression of YBX1) + (0.18925 × expression of NOP2) in the training (TCGA) and validation (GSE65904) dataset.

To further investigate the prognostic value of the risk score, CM patients in the TCGA were classified into high- or low-risk groups according to the median. The results demonstrate that patients with high-risk scores were correlated with a higher death rate (Figure 4(b)). Additionally, we found that the difference between high- and low-risk groups in OS and PFS was significant (Figures 4(c) and 4(d)). Moreover, to validate the predictive efficiency of the prognostic risk score, we used the same analysis in the GSE65904 dataset; the results were in line with the aforementioned findings (Figures 4(e)–4(g)). Our findings suggested that the signature based on five m5C regulators could function as a potent prognostic signature and effectively stratify CM patients based on risk scores providing new insight into targeted therapy.

### 3.6. Relationship between the Risk Score and Clinical Characteristics in CM Patients

**Figure 5:**
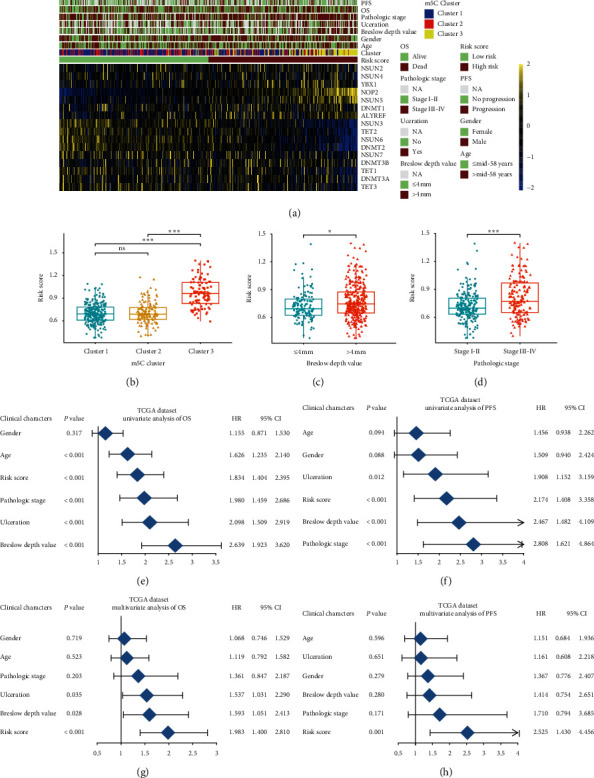
The relationship between the risk score and clinical characteristics. (a) The relationship between sixteen m5C expression profiles stratified by risk score and clinical characteristics in CM patients. (b–d) The relationships between the risk scores and clinical characteristics including m5C clusters, Breslow depth value, and pathologic stage. (e, g) The risk score is an independent prognostic factor of OS in the TCGA dataset. (f, h) The risk score is an independent prognostic factor of PFS in the TCGA dataset.

Next, we tested the correlation of m5C-related genes and risk scores with clinical characteristics. Heatmaps represented the expression of m5C regulators and clinical features in low- and high-risk patients in the TCGA dataset (Figure 5(a)). Results illustrated that high expression of NOP2 and USUN5 and low expression of USUN6, DNMT2, TET2, and USUN3 were associated with the high-risk score. In addition, we analyzed the association between the risk scores and each clinicopathological characteristic. As shown in Figure 5, the risk scores of cluster 2 and cluster 3 were significantly different in the Breslow depth value and pathologic stage (Figures 5(b)–5(d) and Table S5). Moreover, univariate and multivariate Cox regression analyses of OS and PFS in CM were conducted, indicating that the risk score was an independent predictor of prognosis and progression (Figures 5(e)–5(h) and Table S6). Results revealed that the risk scores were significantly correlated with the malignancy of CM.

### 3.7. Importance of m5C Regulators to the Risk Score Using Machine Learning

**Figure 6:**
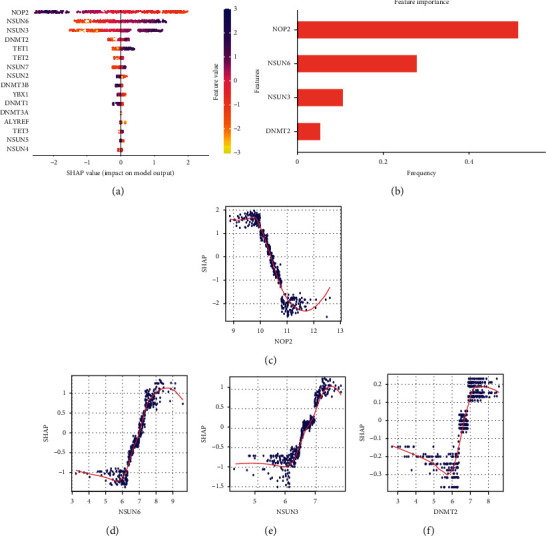
Importance of m5C regulators to the risk score by machine learning. (a) The importance of m5C regulators to the risk score by XGBoost algorithm. (b) The top 4 (NOP2, NSUN6, NSUN3, and DNMT2) important regulators evaluated by the SHAP value. (c–f) The individual SHAP value of NOP2, NSUN6, NSUN3, and DNMT2.

To build a classifier that could recognize the outstanding contributors of risk score, we applied the XGBoost algorithm to build the model. NOP2, NSUN6, NSUN3, and DNMT2 were the top four important regulators evaluated by SHapley additive exPlanation value (SHAP), as shown in Figures 6(b)–6(f). Each point is the feature value of the specific gene. The features are sorted by the sum of the values of all samples. We found that NOP2 and NSUN6 were the most important regulators in our risk score.

### 3.8. Close Correlation of CM Progression and Low Expression of USUN6

**Figure 7:**
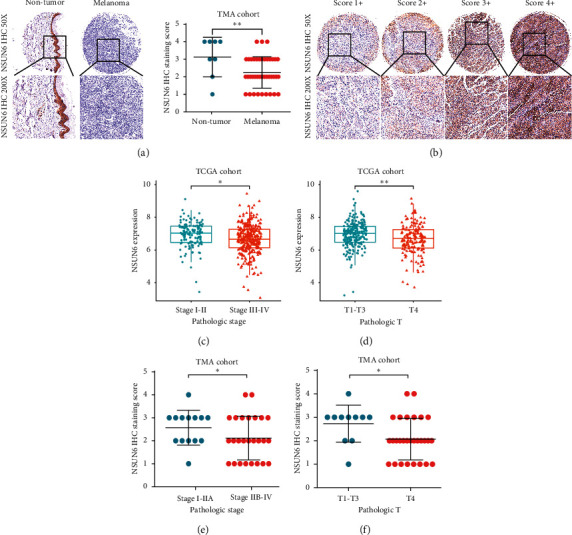
Low expression of USUN6 was closely correlated with CM progression. (a) Decreased expression of USUN6 in CM tissues. (b) Representative images of USUN6 staining in CM tissues. (c–f) The correlation between USUN6 expression levels and the pathologic state and TNM staging of CM.

Subsequently, immunohistochemical staining revealed the decreased protein expression level of NSUN6 in CM tissues which was consistent with our findings (Figure 7(a)). According to the staining intensity, NSUN6 staining was scored from 1+ to 4+ (Figure 7(b)). Furthermore, NSUN6 expression was significantly related to the pathologic state and TNM staging of CM in TCGA and tissue microarray cohorts (Figures 7(c)–7(f)). These findings strongly suggested that USUN6 might serve as a tumor suppressor molecule in CM.

## 4. Discussion

CM is the primary skin cancer mortality cause worldwide owing to its high metastasis, invasiveness, and annually increasing incidence [1, 5]. Although present studies have shown that many complex multistep processes contribute to the initiation, progression, and metastasis of CM, its pathogenesis has not been well determined and effective prognostic biomarkers in CM are still lacking [4]. Therefore, it is valuable to understand the underlying molecular mechanisms, which contribute to the prognostic prediction and therapeutic target of CM. In recent years, m5C modification has been found to play a significant role in the biological function of many cancers. Additionally, the dysregulation of m5C regulators has been observed in numerous cancers including breast, gallbladder, and bladder cancer [25, 26, 28, 33]. Xue et al. [27] recently clarified the m5C-related regulators are more abundant in head and neck squamous cell carcinoma and play vital roles in tumor progression. However, the potential tumorigenesis mechanism and prognosis of CM by dysregulated m5C-related regulators is still elusive.

Our study systematically analyzed the mRNA expressions of m5C regulators and characterized the mutations and copy number variations (CNVs) of m5C regulators in CM for the first time, utilizing data extracted from the public databases. We found that the mRNA expression levels of NOP2, YBX1, DNMT3A, and DNMT1 were significantly increased and NSUN6, NSUN7, TET2, and TET3 were significantly decreased in CM tissues. Somatic mutations and CNV alterations indicated a high mutation frequency of the m5C regulatory gene in CM. Previous studies have demonstrated that some m5C regulators participate in the malignant progression of cancer such as NOP2 and YBX1. The literature suggests that NOP2 is highly expressed in prostate cancer, gallbladder cancer, and lung adenocarcinoma, while upregulated YBX1 plays a significant protumourigenic role in breast, renal, and gastric cancer [34–38]. Conversely, NSUN6 confers cellular fitness advantages and functions as a tumor suppressor in pancreatic cancer [39]. Furthermore, survival analysis of OS and PFS in the TCGA revealed that the m5C regulators had a potential value in evaluating prognosis in CM. In summary, our findings showed that dysregulated m5C regulators may play a key role in the initiation and progression of CM.

Accumulating evidence indicated that m5C RNA methyltransferase-related regulators were implicated in tumorigenesis and tumor development in ovarian cancer, cervical cancer, prostate cancer, glioma, and other several tumors [12, 40]. However, the current study did not investigate the relationship between m5C modifications and CM development. We investigated the association between m5C regulators, clinical features, and prognosis in CM for a comprehensive analysis, finding that m5C regulators had frequent crosstalk. Additionally, we analyzed the expression profiles of m5C regulators, and three clusters with different clinical features and prognoses were identified. Furthermore, we chose hallmark pathways to conduct an in-depth research. Consistent with our results, UV radiation is the primary environmental driver including oxidative stress and DNA damage. Following UV exposure, keratinocytes in a p53-dependent manner produced *α*-melanocyte-stimulating hormone and stimulated the melanocortin 1 receptor to produce melanin. Meanwhile, it has been demonstrated that Wnt/*β*-catenin signaling and angiogenesis played a crucial role in the pathogenesis and progression of CM [41–44]. Therefore, further studies should be conducted on m5C regulators and cancer-promoting signaling pathways that form a potential comprehensive network, significantly influencing CM progression.

We constructed a risk score, including the expression of DNMT2, NSUN3, NSUN6, YBX1, and NOP2, using the scoring algorithm in the TCGA (training set) and the GSE65904 (verification set) to accurately evaluate the patient's prognosis. Based on the median, CM patients were divided into high- and low-risk groups. Further analysis revealed that risk score had suitable stratification abilities for predicting prognosis and tumor progression in CM patients. Moreover, the risk score could independently predict prognosis and progression in CM patients. Moreover, the high-risk group is highly correlated with the advanced pathologic stage and deeper Breslow depth. These results demonstrate that the m5C regulators might be regarded as a potential tool to predict progression and prognosis in CM patients.

To gain more information on the core signature among m5C regulators in CM, we performed machine learning via the XGBoost algorithm [45–48]. An interesting finding was that, of m5C regulators, NOP2 contributed the most to CM, while NSUN6 ranked second. NOP2 (also named NSUN1, p120) has been improved primarily for its protumorigenic roles in many cancers such as prostate cancer, gallbladder cancer, lung adenocarcinoma, and several other cancers, consistent with our study [34, 49, 50]. NSUN6 targets mRNA to transfer methyl and is higher expressed in healthy tissues than in tumors. Previous studies also found that NSUN6 could inhibit pancreatic cancer development [39, 51]. However, the role of NSUN6 in CM has not been documented thus far. To our best knowledge, our study would be a pioneering contribution to the suppressive role of NSUN6 in CM. Finally, using immunohistochemistry analysis, we found that the expression level of NSUN6 was lower in CM tissues than in the corresponding peri-CM tissues. In summary, our studies revealed that NSUN6 was a significant contribution suppressor molecule in CM.

## 5. Conclusions

We explored the relationship between m5C regulators and the progression and prognosis of CM. A risk score model was also established and validated to predict the progression and prognosis of CM patients. Machine learning algorithms indicated that NSUN6 was a significant contribution suppressor molecule in CM. Our results provide a unique approach to the application of novel diagnostic biomarkers and targeted therapy for CM.

## Data Availability

The data used to support the findings of this study are included within the article.

## Supplementary Materials

Supplementary Materials Table S1: the characteristics of public databases used in this study. Table S2: demographics of the cohort. Table S3: information on antibodies used in this study. Table S4: clinical characteristics of patients with m5C clusters in the TCGA cohort. Table S5: clinical characteristics of patients with m5C risk score in the TCGA cohort. Table S6: the univariate and multivariate Cox regression analysis of m5C regulators, risk score, and clinical features in the TCGA cohort. Figure S1: Kaplan–Meier analysis of OS of sixteen m5C regulators. Figure S2: Kaplan–Meier analysis of PFS of sixteen m5C regulators. Figure S3: unsupervised consensus analysis of sixteen m5C regulators. (a–e) Consensus clustering matrix for *k* = 2, *k* = 3, *k* = 4, *k* = 5, and *k* = 6. (f) Consensus clustering cumulative distribution function for *k* = 2 to 6. (Supplementary Materials).
