# Supplementary material for: m5C-Related Signatures for Predicting Prognosis in Cutaneous Melanoma with Machine Learning
Source: J Oncol. 2021 Aug 4;2021:6173206. doi: 10.1155/2021/6173206 (PMC8360728; doi:10.1155/2021/6173206)
Supplement: Supplementary Materials — Table S1: the characteristics of public databases used in this study. Table S2: demographics of the cohort. Table S3: information on antibodies used in this study. Table S4: clinical characteristics of patients with m5C clusters in the TCGA cohort. Table S5: clinical characteristics of patients with m5C risk score in the TCGA cohort. Table S6: the univariate and multivariate Cox regression analysis of m5C regulators, risk score, and clinical features in the TCGA cohort. Figure S1: Kaplan–Meier analysis of OS of sixteen m5C regulators. Figure S2: Kaplan–Meier analysis of PFS of sixteen m5C regulators. Figure S3: unsupervised consensus analysis of sixteen m5C regulators. (a–e) Consensus clustering matrix for k = 2, k = 3, k = 4, k = 5, and k = 6. (f) Consensus clustering cumulative distribution function for k = 2 to 6. (Supplementary Materials). [file 6173206.f1.zip › 6173206.f1/Table S2.pdf]

TableS2: Demographics of the cohort

Features		TCGA cohort (n=461)
Age		
	≤58 years	236 (51.19)
	> 58 years	225 (48.81)
Gender		
	Male	286 (62.04)
	Female	175 (37.96)
Breslow depth value		
	≤4mm	202 (43.82)
	> 4mm	154 (33.41)
	NA	105 (22.77)
Ulceration		
	yes	166 (36.00)
	no	146 (31.67)
	NA	149 (32.32)
Pathologic stage		
	stage I-II	124 (26.91)
	stage III-IV	301 (65.29)
	NA	36 (7.80)
