# Supplementary material for: m5C-Related Signatures for Predicting Prognosis in Cutaneous Melanoma with Machine Learning
Source: J Oncol. 2021 Aug 4;2021:6173206. doi: 10.1155/2021/6173206 (PMC8360728; doi:10.1155/2021/6173206)
Supplement: Supplementary Materials — Table S1: the characteristics of public databases used in this study. Table S2: demographics of the cohort. Table S3: information on antibodies used in this study. Table S4: clinical characteristics of patients with m5C clusters in the TCGA cohort. Table S5: clinical characteristics of patients with m5C risk score in the TCGA cohort. Table S6: the univariate and multivariate Cox regression analysis of m5C regulators, risk score, and clinical features in the TCGA cohort. Figure S1: Kaplan–Meier analysis of OS of sixteen m5C regulators. Figure S2: Kaplan–Meier analysis of PFS of sixteen m5C regulators. Figure S3: unsupervised consensus analysis of sixteen m5C regulators. (a–e) Consensus clustering matrix for k = 2, k = 3, k = 4, k = 5, and k = 6. (f) Consensus clustering cumulative distribution function for k = 2 to 6. (Supplementary Materials). [file 6173206.f1.zip › 6173206.f1/Table S3.pdf]

Supplementary Table S2. Information on antibodies used in this study			
**Antibody**	**IHC**	**Specificity**	**Company**
**NSUN6**	1:200	Rabbit Polyclonal	Proteintech Group, China
