# Supplementary material for: m5C-Related Signatures for Predicting Prognosis in Cutaneous Melanoma with Machine Learning
Source: J Oncol. 2021 Aug 4;2021:6173206. doi: 10.1155/2021/6173206 (PMC8360728; doi:10.1155/2021/6173206)
Supplement: Supplementary Materials — Table S1: the characteristics of public databases used in this study. Table S2: demographics of the cohort. Table S3: information on antibodies used in this study. Table S4: clinical characteristics of patients with m5C clusters in the TCGA cohort. Table S5: clinical characteristics of patients with m5C risk score in the TCGA cohort. Table S6: the univariate and multivariate Cox regression analysis of m5C regulators, risk score, and clinical features in the TCGA cohort. Figure S1: Kaplan–Meier analysis of OS of sixteen m5C regulators. Figure S2: Kaplan–Meier analysis of PFS of sixteen m5C regulators. Figure S3: unsupervised consensus analysis of sixteen m5C regulators. (a–e) Consensus clustering matrix for k = 2, k = 3, k = 4, k = 5, and k = 6. (f) Consensus clustering cumulative distribution function for k = 2 to 6. (Supplementary Materials). [file 6173206.f1.zip › 6173206.f1/Table S4.pdf]

Table-S3: Clinical Characteristics of patients with m5C clusters in TCGA cohort				
Variable	Cluster1 N=248	Cluster2 N=118	Cluster3 N=95	P-value
Median age				6.11E-01
<=58years	123	65	48	
>58years	125	53	47	
Gender				6.43E-01
Male	157	74	55	
Female	91	44	40	
Pathologic stage				1.28E-02
I-II	61	43	20	
III-IV	165	65	71	
Uceration				1.10E-01
No	81	38	27	
Yes	90	31	45	
Breslow depth value				5.06E-04
<=4 mm	107	66	29	
>4 mm	77	31	46	

TCGA, the Cancer Genome Atla
