# Supplementary material for: m5C-Related Signatures for Predicting Prognosis in Cutaneous Melanoma with Machine Learning
Source: J Oncol. 2021 Aug 4;2021:6173206. doi: 10.1155/2021/6173206 (PMC8360728; doi:10.1155/2021/6173206)
Supplement: Supplementary Materials — Table S1: the characteristics of public databases used in this study. Table S2: demographics of the cohort. Table S3: information on antibodies used in this study. Table S4: clinical characteristics of patients with m5C clusters in the TCGA cohort. Table S5: clinical characteristics of patients with m5C risk score in the TCGA cohort. Table S6: the univariate and multivariate Cox regression analysis of m5C regulators, risk score, and clinical features in the TCGA cohort. Figure S1: Kaplan–Meier analysis of OS of sixteen m5C regulators. Figure S2: Kaplan–Meier analysis of PFS of sixteen m5C regulators. Figure S3: unsupervised consensus analysis of sixteen m5C regulators. (a–e) Consensus clustering matrix for k = 2, k = 3, k = 4, k = 5, and k = 6. (f) Consensus clustering cumulative distribution function for k = 2 to 6. (Supplementary Materials). [file 6173206.f1.zip › 6173206.f1/Table S5.pdf]

Supplementary Table-S4: Clinical Characteristics of patients with m5C risk score in TCGA cohort

Variable	Low risk N=231	High risk N=230	P-value
Median age			7.45E-01
<=58years	120	116	
>58years	111	114	
Gender			3.08E-01
Male	138	148	
Female	93	82	
Pathologic stage			5.00E-02
I-II	70	54	
III-IV	139	162	
Uceration			1.92E-01
No	75	71	
Yes	73	93	
Breslow depth value			1.04E-04
<=4 mm	118	84	
>4 mm	58	96	
m5C cluster (%)			7.76E-19
Cluster 1	149	99	
Cluster 2	74	44	
Cluster 3	8	87	
