# Supplementary material for: m5C-Related Signatures for Predicting Prognosis in Cutaneous Melanoma with Machine Learning
Source: J Oncol. 2021 Aug 4;2021:6173206. doi: 10.1155/2021/6173206 (PMC8360728; doi:10.1155/2021/6173206)
Supplement: Supplementary Materials — Table S1: the characteristics of public databases used in this study. Table S2: demographics of the cohort. Table S3: information on antibodies used in this study. Table S4: clinical characteristics of patients with m5C clusters in the TCGA cohort. Table S5: clinical characteristics of patients with m5C risk score in the TCGA cohort. Table S6: the univariate and multivariate Cox regression analysis of m5C regulators, risk score, and clinical features in the TCGA cohort. Figure S1: Kaplan–Meier analysis of OS of sixteen m5C regulators. Figure S2: Kaplan–Meier analysis of PFS of sixteen m5C regulators. Figure S3: unsupervised consensus analysis of sixteen m5C regulators. (a–e) Consensus clustering matrix for k = 2, k = 3, k = 4, k = 5, and k = 6. (f) Consensus clustering cumulative distribution function for k = 2 to 6. (Supplementary Materials). [file 6173206.f1.zip › 6173206.f1/Table S6.pdf]

Table S6: The univariate and multivariate Cox regression analysis of m5C regulators, risk score and clinical features in TCGAcohort

TCGA univariate analysis of OS						TCGA univariate analysis of PFS					
m5C regulators	p.val	HR	lower	upper	p.adjust	m5C regulators	p.val	HR	lower	upper	p.adjust
NSUN3	0.001	0.664	0.520	0.849	0.005	NSUN6	0.385	0.887	0.677	1.162	0.880
DNMT2	0.000	0.774	0.666	0.899	0.001	NSUN3	0.744	0.933	0.617	1.411	0.961
NSUN6	0.002	0.779	0.667	0.910	0.008	NSUN7	0.277	0.934	0.825	1.057	0.880
NSUN7	0.181	0.950	0.880	1.024	0.386	YBX1	0.852	0.960	0.627	1.471	0.961
TET2	0.555	0.964	0.855	1.088	0.745	TET3	0.794	0.964	0.735	1.266	0.961
NSUN2	0.845	0.972	0.731	1.293	0.901	DNMT2	0.881	0.980	0.758	1.268	0.961
TET1	0.943	0.996	0.889	1.115	0.943	TET1	0.852	0.982	0.812	1.188	0.961
DNMT3A	0.817	1.022	0.847	1.234	0.901	NSUN4	0.995	1.001	0.633	1.584	0.995
TET3	0.559	1.051	0.889	1.244	0.745	DNMT3A	0.901	1.019	0.754	1.379	0.961
NSUN4	0.679	1.067	0.786	1.448	0.836	TET2	0.537	1.066	0.870	1.305	0.961
DNMT3B	0.193	1.083	0.961	1.220	0.386	ALYREF	0.551	1.108	0.792	1.550	0.961
DNMT1	0.438	1.090	0.877	1.355	0.701	DNMT3B	0.164	1.141	0.948	1.373	0.875
ALYREF	0.252	1.128	0.918	1.386	0.448	DNMT1	0.380	1.176	0.819	1.688	0.880
NSUN5	0.121	1.176	0.958	1.445	0.323	NSUN2	0.219	1.330	0.844	2.096	0.876
YBX1	0.038	1.327	1.015	1.734	0.122	NSUN5	0.053	1.408	0.996	1.992	0.424
NOP2	0.000	1.476	1.196	1.820	0.001	NOP2	0.002	1.696	1.220	2.358	0.032
**TCGA univariate analysis of OS**						**TCGA univariate analysis of PFS**					
gender	0.317	1.155	0.871	1.530	0.317	age	0.094	1.456	0.938	2.262	0.094
age	0.001	1.626	1.235	2.140	0.001	gender	0.088	1.509	0.940	2.424	0.094
risk score	<0.001	1.834	1.404	2.395	<0.001	ulceration	0.012	1.908	1.152	3.159	0.018
pathologic.stage	<0.001	1.980	1.459	2.686	<0.001	risk score	0.001	2.174	1.408	3.358	0.002
ulceration	<0.001	2.098	1.509	2.919	<0.001	breslow.depth.value	0.000	2.467	1.482	4.109	0.000
breslow.depth.value	<0.001	2.639	1.923	3.620	<0.001	pathologic.stage	0.000	2.808	1.621	4.864	0.000
**TCGA multivariate analysis of OS**						**TCGA multivariate analysis of PFS**					
gender	0.719	1.068	0.746	1.529	0.719	age	0.596	1.151	0.684	1.936	0.651
age	0.523	1.119	0.792	1.582	0.628	ulceration	0.651	1.161	0.608	2.218	0.651
pathologic stage	0.203	1.361	0.847	2.187	0.305	gender	0.279	1.367	0.776	2.407	0.420
ulceration	0.035	1.537	1.031	2.290	0.070	breslow depth value	0.280	1.414	0.754	2.651	0.420
breslow depth value	0.028	1.593	1.051	2.413	0.070	pathologic stage	0.171	1.710	0.794	3.685	0.420
risk score	0.001	1.983	1.400	2.810	0.006	risk score	0.001	2.525	1.430	4.456	0.006
